# P09 - Meta-analysis: spectrum of HDM allergic rhinitis and asthma in Malaysia

**DOI:** 10.1186/2045-7022-4-S1-P64

**Published:** 2014-02-28

**Authors:** Davendralingam Sinniah, Subha Sethu Thakachy

**Affiliations:** 1 International Medical University Medical School Seremban, Seremban, Malaysia

## Introduction

Asthma prevalence is increasing worldwide. Its incidence is highest in the West and less common in developing countries. Allergic rhinitis and asthma prevalence is increasing in Malaysia as it progresses towards developed nation status. Careful assessment of clinical history and symptoms provide clues to the causative allergen but more definitive data is needed on the aetiological agents.

## Objective

This paper reviews the spectrum of HDM related AR and asthma in Malaysia based on results of SPT.

## Method

A Google Scholar search was undertaken of all published literature on HDM allergy in Malaysia.

## Results

A total of 14 papers comprising 7 on AR, and 6 on asthma were published during the years 1992 to 2011. In the 7 studies comprising1563 patients with AR, SPT for HD was positive in a mean of 71% (range 60 – 86%) of cases. In the 6 studies on asthma comprising 380 patients, the SPT for HDM was positive in a mean of 76% (range 50 – 90%) of cases. In another study (ref), positive SPTs with mite extracts were detected in more than 80% of asthmatic and rhinitis patients. Several HDM surveys^[2,3,7]^ suggested that *Dermatophagoides* species are an important source for the allergic sensitisation, but *Blomia tropicalis* has recently been identified as the most common and abundant species of HDM^[4]^. Thirty five percentage of patients’ seeking treatment at Otorhinolaryngology Clinics in Malaysia suffer from allergic rhinitis. Allergic rhinitis and asthma often coexist.

## Conclusion

There is increasing prevalence of AR and asthma associated with HDM sensitivity in Malaysia and the region. This is attributed to improvement in socioeconomic conditions, Westernized life styles and home environments conducive to proliferation of HDM and related HDM sensitivity.

